# EcoliNet: a database of cofunctional gene network for *Escherichia coli*

**DOI:** 10.1093/database/bav001

**Published:** 2015-02-02

**Authors:** Hanhae Kim, Jung Eun Shim, Junha Shin, Insuk Lee

**Affiliations:** Department of Biotechnology, College of Life Science and Biotechnology, Yonsei University, Seoul, Korea

## Abstract

During the past several decades, *Escherichia coli* has been a treasure chest for molecular biology. The molecular mechanisms of many fundamental cellular processes have been discovered through research on this bacterium. Although much basic research now focuses on more complex model organisms, *E. coli* still remains important in metabolic engineering and synthetic biology. Despite its long history as a subject of molecular investigation, more than one-third of the *E. coli* genome has no pathway annotation supported by either experimental evidence or manual curation. Recently, a network-assisted genetics approach to the efficient identification of novel gene functions has increased in popularity. To accelerate the speed of pathway annotation for the remaining uncharacterized part of the *E. coli* genome, we have constructed a database of cofunctional gene network with near-complete genome coverage of the organism, dubbed EcoliNet. We find that EcoliNet is highly predictive for diverse bacterial phenotypes, including antibiotic response, indicating that it will be useful in prioritizing novel candidate genes for a wide spectrum of bacterial phenotypes. We have implemented a web server where biologists can easily run network algorithms over EcoliNet to predict novel genes involved in a pathway or novel functions for a gene. All integrated cofunctional associations can be downloaded, enabling orthology-based reconstruction of gene networks for other bacterial species as well.

**Database URL**: http://www.inetbio.org/ecolinet

## Introduction

*Escherichia coli* is perhaps the most intensively studied species of bacteria, due to its utility in both exploring the molecular mechanisms underlying fundamental biological processes and manufacturing useful metabolites for the biomedical industry. Numerous molecular genetics techniques have been developed in *E. coli* over the past several decades, making it the standard bacterial species in which to study genetics and the molecular mechanisms underlying cellular phenotypes. This attention has led to the elucidation of many conserved metabolic pathways in *E. coli*, resulting in its use as a metabolic engineering platform. Despite its importance in science and engineering, a significant portion of the *E. coli* genome remains uncharacterized. For example, as of September 2014, the Gene Ontology database (1) had no biological process annotations supported by either experimental evidence or manual curation for ∼2000 protein coding genes. A holistic *E. coli* pathway map could significantly improve our ability to engineer metabolic phenotypes by providing a genetic circuit design that accounts for the entire system.

Although traditional forward and reverse genetic approaches have played major roles in gene-to-phenotype association mapping in *E. coli*, a more efficient and sensitive genetics approach would facilitate characterization of the part of the genome whose function is not yet known. Network-assisted predictive genetics is an example of such an approach whose popularity is growing (2, 3). Here, we present a functional gene network for *E. coli*, dubbed EcoliNet, which includes 95 520 cofunctional associations and covers ∼99% of the genome. EcoliNet has high predictive power for a wide variety of bacterial phenotypes, including response to various stresses and drugs. To make EcoliNet freely available as a hypothesis-generating tool, we have implemented a web server where users can conduct network algorithms, prioritizing novel genes for a pathway or novel functions for an *E. coli* gene. The EcoliNet server (http://www.inetbio.org/ecolinet) provides not only public prediction tools but also a database of cofunctional associations between *E. coli* genes, derived from diverse biological data. Moreover, cofunctional gene networks for other bacterial species can be constructed via orthology-based transfer of information from EcoliNet.

## Construction of cofunctional networks

Cofunctional links between *E. coli* genes were inferred from seven distinct types of data as summarized in Table 1. The inferred links with data-intrinsic measures were benchmarked using gold standard gene pairs derived from annotation databases for pathways and biological processes. Hence, two genes linked by significant benchmarking score are likely to operate same pathways or biological processes. The database contains seven cofunctional networks derived from each of seven data types and an integrated network. The network construction follows a machine learning process with Bayesian statistics framework. More details about the network construction are described below.

**Table 1.** Seven distinct types of data incorporated into EcoliNet

Code	Data type description	# unique genes	# unique gene pairs
CC	Cofunctional links inferred from cocitation of two genes across 57 062 PubMed Central (PMC) articles for *E. coli* biology	2296	50 528
CX	Cofunctional links inferred from coexpression pattern of two *E. coli* genes (based on high-dimensional gene expression data)	4039	67 494
DC	Cofunctional links inferred from co-occurrence of protein domains between two *E. coli* coding genes	2283	9643
GN	Cofunctional links inferred from similar genomic context of bacterial orthologs of two *E. coli* genes	3568	23 439
HT	Cofunctional links inferred from high-throughput protein-protein interactions between two *E. coli* genes	3209	15 543
LC	Cofunctional links inferred from small/medium-scale protein-protein interactions (collected from protein-protein interaction data bases) between two *E. coli* genes	764	1073
PG	Cofunctional links inferred from similar phylogenetic profiles between two *E. coli* genes	1817	17 504
EcoliNet	A cofunctional *E. coli* gene network by integration of all above link sets	4099	95 520

### *E*. *coli* genome data

*E. coli* genome was downloaded from National Center for Biotechnology Information genome database (ftp://ftp.ncbi.nlm.nih.gov/genomes/) on 11 November 2011. It has a total of 4496 genes, of which 4146 protein coding genes were used to construct EcoliNet. For functional annotations, we used Gene Ontology (1) downloaded on February 2013 and EcoCyc, version 16.5 (4).

### Gold-standard cofunctional gene pairs for network training

A functional gene network is constructed through a machine learning process. A gold-standard set of gene associations works as a cornerstone for error-tolerant and unbiased learning. To construct gold-standard data, we generated positive cofunctional links by pairing genes that share at least one annotation by Gene Ontology Biological Process (GO-BP) terms with GO evidence code of IDA (inferred from direct assay), IGI (inferred from genetic interaction) or IMP (inferred from mutant phenotype). Five GO-BP terms with the largest number of member genes—DNA-dependent transcription (GO:00006351), DNA-dependent negative regulation of transcription (GO:0045892), DNA-dependent regulation of transcription (GO:0006355), DNA-dependent phosphorelay signal transduction system (GO:0000160), DNA-dependent positive regulation of transcription (GO:0045893)—were excluded to avoid biased learning towards these large biological processes (5). After this filtration, 6896 cofunctional associations among 1474 genes were derived from GO-BP annotations. In addition, we employed *E. coli* pathway annotations by EcoCyc and MetaCyc (6). With exclusion of superpathways to avoid between-pathway associations, we obtained 4694 cofunctional links among 885 genes from those annotations. EcoCyc and MetaCyc provide highly redundant information and provide only 347 and 193 complementary links, respectively. Only 786 of the 4694 links (17%), however, were overlap with GO-BP links. Therefore, GO-BP, EcoCyc and MetaCyc together provide a total of 10 804 positive gold standard functional associations among 1835 genes. We also inferred 1 671 891 negative gold-standard functional associations by connecting 2 of the 1835 annotated genes that do not share any of the annotations.

### Probabilistic integration of cofunctional links

Using Bayesian statistical framework, we devised log-likelihood scores (*LLS*) to measure likelihood of a functional association between two genes for the given supporting evidence (7). For given gold-standard cofunctional link (L) and supporting evidence (E), *LLS* is represented as:

$$LLS=\text{ln}\left(\frac{P\left(L|E\right)/P\left(∼L|E\right)}{P\left(L\right)/P\left(∼L\right)}\right)$$

whereP(L|E) and P(∼L|E) are the frequencies of positive and negative gold-standard links in condition of the given evidence, respectively, whereas P(L) and P(∼L) are the frequencies of all positive and all negative gold-standard links, respectively. All individual sets of gene pairs inferred from different evidences are scored by the LLS.

To increase completeness of a network model, all individual linkage sets inferred from different evidences are integrated based on the unified LLS. Due to the occurrence of a functional association supported by multiple evidences, we devised a weighted sum (WS) method which is a variant of naïve Bayesian integration (5). Unlike naïve Bayesian integration, the WS effectively handle correlation among data sets during integration. For example, in summation of LLS from multiple data sets, the WS method reduces redundant information by adjusting weight for different data sets by the following equation:

$$\text{WS}=S_{0}+\overset{n}{\underset{i=1}{\sum }}\frac{S_{i}}{D\times i},\;for\;all\;S\geq T,$$

where S is LLS for the given cofunctional link, and *i* is the rank index of LLS; $S_{0}$ is the best LLS. A free parameter D is a weight factor, and *T* is the cutoff of minimum LLS to be integrated.

### Cofunctional links inferred from cocitation (*CC*)

Cofunctional genes tend to appear in the same article (8). By scanning full texts of 57 062 articles with abstract containing the word ‘*Escherichia coli*’ or ‘*E. coli*’ in the PubMed Central database, we collected pair relationship between an *E. coli* gene name and an article. Then we measure significance of CC between two genes using hypergeometric probability.

### Cofunctional links inferred from coexpression (*CX*)

We collected 132 microarray series containing more than 8 expression samples from Gene Expression Omnibus (GEO) at March 2013 (9). A functional association of two genes can be inferred by coexpression patterns across given experimental conditions using Pearson correlation coefficient. We observed significant correlation between coexpression and cofunctional association from 54 series containing a total of 1709 samples. We integrated the 54 coexpression networks derived from the 54 series into a single coexpression network using WS method. This integrated network was then integrated into the EcoliNet. All 54 coexpression networks are downloadable from EcoliNet server.

### *Cofunctional links inferred from the cooccurrence of protein domains* (*DC*)

Protein domains are recurring sequence units of protein region, involved in protein function and evolution. Cofunctional genes often share the same protein domain. Thus, we may be inferred functional association by significant domain cooccurrence between two proteins based on InterPro database (10). We measure significance of domain cooccurrence between two proteins by weighted mutual information (WMI), which accounts for frequency of each domain (11). In the WMI, rarer domains receive higher weights assuming that rarer domains are associated to more specific pathways.

### Cofunctional links inferred from genomic contexts (*GN and PG*)

Similar genomic contexts between two genes may reflect their functional couplings under evolutionary and regulatory constraints. We employed two effective genomic context approaches, phylogenetic profile similarity (PG) (12–14) and gene neighborhood (GN) (15–17), to infer functional associations between two genes. We used a total of 1748 fully sequenced prokaryotic genomes (122 for Archaea, 1626 for Bacteria). The similarity of the genomic contexts during speciation can be measured by coinheritance patterns of two genes in phylogenetic profiles. We, first, ran all *E. coli* protein coding sequences against all protein coding sequences of the 1748 genomes to obtain the similarity profile matrices of the genomic contexts by calculating blast hit scores. The generated profile matrices were used to calculate mutual information scores as for Date *et al.* (18). For EcoliNet, we built two networks specific to each of two domains, Archaea and Bacteria, and then integrated them into a single phylogenetic profile network. To infer cofunctional association by GN, we used two complementary methods of measuring genomic neighborhood: distance-based GN and probability-based GN (19). These two networks were then integrated into a single GN network.

### Cofunctional links inferred from protein–protein interactions (*HT and LC*)

Functional associations between genes can be inferred from two types of protein–protein interaction (PPI) data sources: (i) small/medium scale PPIs (LC) derived from curated PPI databases such as IntAct (20), DIP (21), MINT (22), BioGRID (23); (ii) large-scale high-throughput PPIs (HT) inferred by affinity purification-mass spectrometry (24–26) and yeast two hybrid analysis (27). These four networks inferred from high-throughput and literature curation PPIs are downloadable from the EcoliNet server.

## Network assessment and applications

### Assessment of EcoliNet

**Figure 1.**
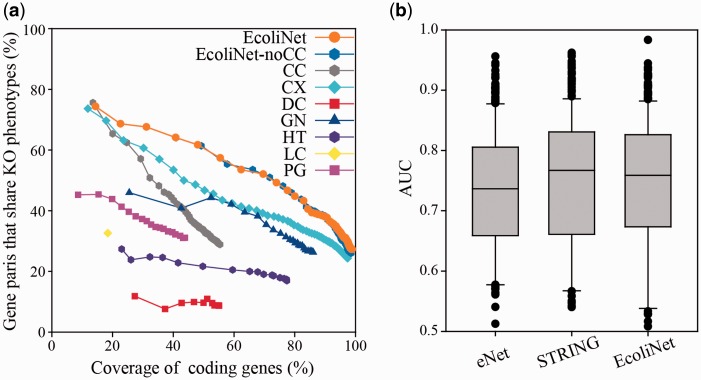
(**a**) Assessment of *E. coli* gene networks performed using data on knockout phenotypes in the presence of 324 different growth and chemical stress conditions. The plot represents the percentage coverage of the *E. coli* coding genome vs. the percentage of *E. coli* gene pairs that share at least one knockout (KO) phenotype. Cumulative accuracy (*y*-axis) of a network was measured for each successive bin of 1000 gene pairs sorted by network edge scores, and coverage (*x*-axis) of each accuracy measure was based on the total number of genes of the cumulated set of gene pairs. EcoliNet outperforms all individual networks derived from seven data types as well as eNet. EcoliNet with no links derived from CCs (EcoliNet-noCC) shows overlap of performance curves for the assessment with the intact EcoliNet. Codes for seven data types are summarized in Table 1. (**b**) Box-and-whisker plots summarize the prediction performance for eNet, STRING, and EcoliNet. The AUC values between eNet and EcoliNet shows significant difference (*P* = 2.28 × 10^−4^, Wilcoxon signed rank test), while those between STRING and EcoliNet are not significantly different (*P* = 0.31, Wilcoxon signed rank test). Because the number of links can affect performance measure, we used only top 79 876 links, the size of eNet, for testing all three networks.

EcoliNet comprises 4099 *E. coli* coding genes (∼99% of the coding genome) and 95 520 cofunctional links. Fair evaluation of trained cofunctional links requires a validation set of gene pairs that are independent from the original training set for the links. To assess the predictive power of EcoliNet, we used data from knockout phenotypes subjected to 324 growth and chemical stress conditions (28). We defined genes associated with each stress phenotype as those with a growth defective score below −4, then measured the percentage of gene pairs that shared at least one relevant stress phenotype. Network accuracy for the given coverage of coding genes shows that the integrated network, EcoliNet, outperforms networks derived from individual component data types (Figure 1a), demonstrating effectiveness of the data integration in constructing a genome-scale network for *E. coli*. To test whether potentially circular logic derived from literature-based network information resulted in the overestimation of EcoliNet’s performance, we also assessed EcoliNet in the absence of links derived from only CCs (EcoliNet-noCC). We found no performance difference for the assessment after removal of cofunctional gene pairs inferred from only cocitation, indicating that circular logic did not compromise our validation and that the measured predictive power of EcoliNet can be generalized to many other phenotypes.

We also compared EcoliNet to other widely used cofunctional *E. coli* gene networks, eNet (26) and STRING (29), for the phenotype prediction. We measured network prediction power for each knockout phenotype with leave-one-out analysis setting in which a gene for a knockout phenotype is prioritized by network connections to all other member genes. Retrieval rate of true predictions for each knockout phenotype was measured by receiver operating characteristic (ROC) analysis, which is summarized as area under the ROC curve (AUC) score. We found no significant difference between EcoliNet and STRING (*P* = 0.31, Wilcoxon signed rank test), while EcoliNet showed significantly higher performance than eNet (*P *= 2.28 × 10^−^^4^, Wilcoxon signed rank test) in phenotype prediction power (Figure 1b). Although EcoliNet and STRING are similar in prediction power, EcoliNet has a merit of hypothesis generation service. The EcoliNet web server provides interactive web interface in which users can generate candidate gene-to-phenotype hypotheses for query genes, while STRING allows only browsing interacting proteins of query genes.

### Public data and utilities of EcoliNet

**Figure 2.**
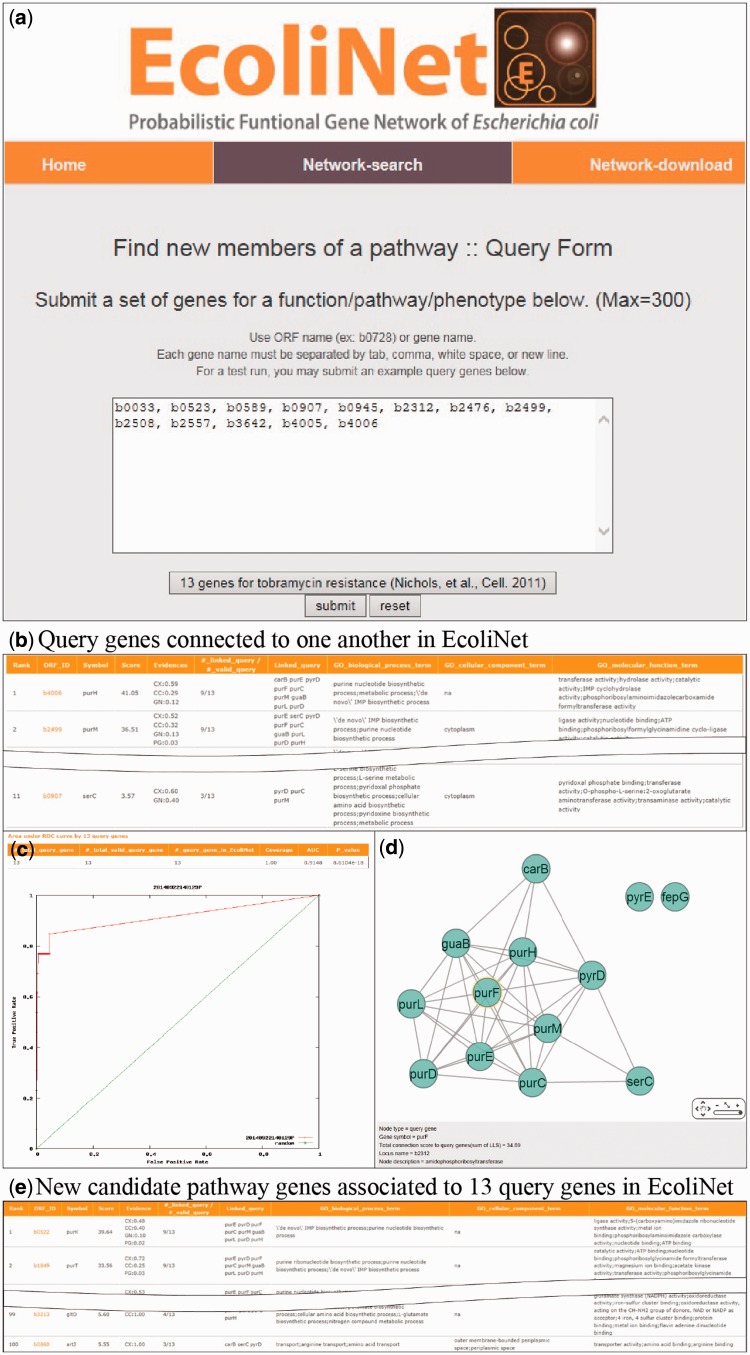
EcoliNet search results by ‘Find new members of a pathway’ option with 13 query genes for tobramycin resistance. (**a**) ‘Find new members of a pathway’ submission page generally takes multiple query genes with ORF name or gene name. (**b**) All connected query genes (11 out of 13 query genes) in EcoliNet are listed in a table. (**c**) ROC curve analysis results in a high AUC score (0.915), which indicates that known tobramycin resistance genes can be highly predictable by connections among them in EcoliNet. (**d**) A network of query genes is visualized by Cytoscape Web installed in EcoliNet web server. (**e**) New candidate genes for tobramycin resistance are prioritized by sum of edge weight scores (log likelihood score) to all query genes and top 100 candidate genes are listed in a table with various information such as data types supporting association with the query genes.

**Figure 3.**
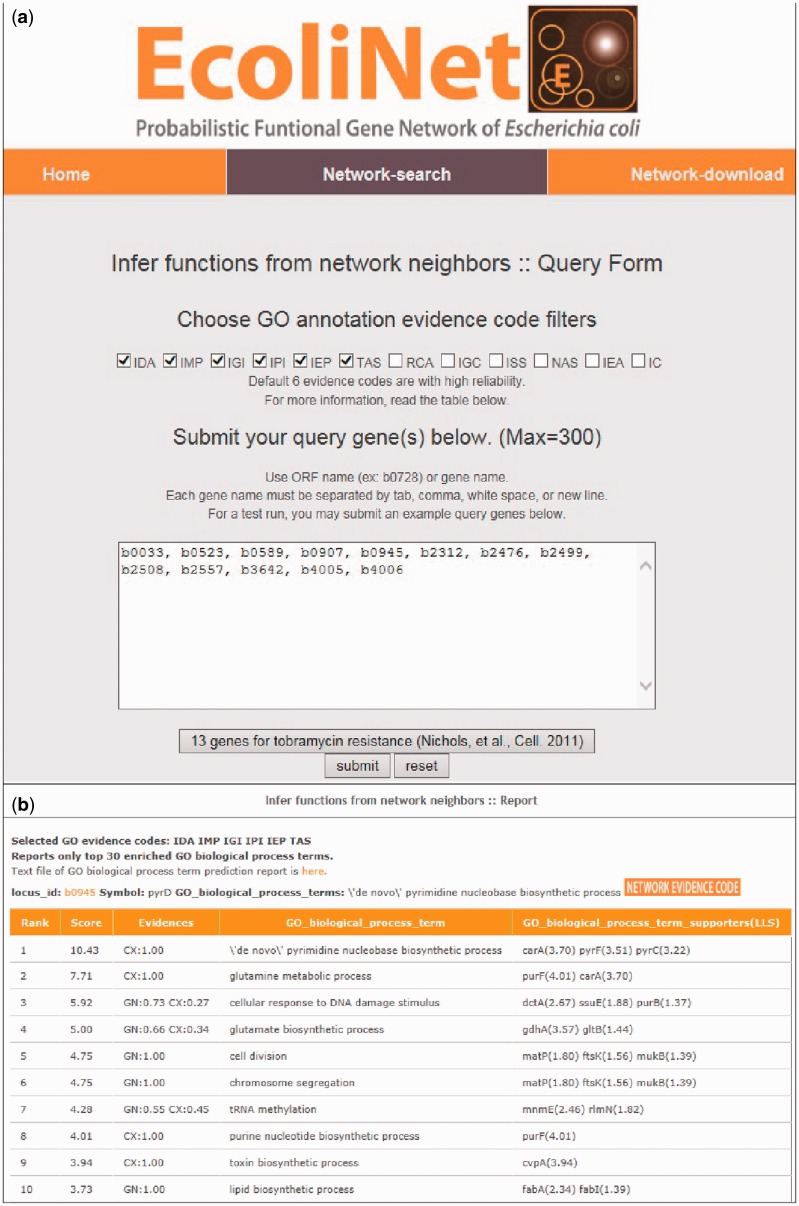
EcoliNet search results by ‘Infer functions from network neighbors’ option for tobramycin resistance genes. (**a**) ‘Infer fucntions from network neighbors’ option may take multiple query genes. GO-BP terms for prediction can be filtered for various GO evidences and default setting used the following six types of reliable evidences: inferred from direct assay (IDA), inferred from mutant phenotype (IMP), inferred from genetic interaction (IGI), inferred from physical interaction (IPI), inferred from expression pattern (IEP), traceable author statement (TAS). (**b**) Top 10 inferred GO-BP terms for ‘b0945’, a genes for tobramycin resistance.

To maximize EcoliNet’s usability, we have implemented a web server where experimental biologists can run network algorithms to predict novel candidate genes for a given phenotype (Figure 2) or novel candidate functions for a gene of interest (Figure 3). If a user submits genes that show similar knockout phenotypes to ‘Find new members of a pathway’ query submission page (Figure 2a), search function first analyzes the connectivity among those genes using ROC analysis, which is summarized as area under the curve (AUC) score, and visualizes their network using Cytoscape Web software (30) installed on EcoliNet server. For example, Figure 2b–e shows results from ‘Find new members of a pathway’ search using 13 *E. coli* query genes whose null mutants show increased resistance against tobramycin. A high AUC score (Figure 2c) and appearance of a highly connected network (Figure 2d) for the query genes indicate that most intrinsic tobramycin resistant genes are functionally coupled and that other associated genes could be new candidates for tobramycin resistant genes. Hence, the server provides the top 100 genes connected to the 13 tobramycin resistance genes submitted, as new candidates (Figure 2e). If a user submits uncharacterized genes to ‘Infer functions from network neighbors’ query submission page (Figure 3a), the server provides the top 30 candidate GO-BP terms for each query gene (Figure 3b). The user may choose specific GO evidence codes for functional prediction; the default setting uses only GO-BP terms supported by experimental evidence and the literature.

EcoliNet freely distributes edge information, not only for the integrated network but also for all individual component networks, including those for individual data sets at network download page of the EcoliNet web server (http://www.inetbio.org/ecolinet/downloadnetwork.php). These data will allow alternative network integration, which can be used to construct new *E. coli* gene networks. Moreover, orthology-based network transfer enables construction of gene networks for other bacterial species, including many pathogens (31).

## Discussion

Although *E. coli* is one of the most intensively studied and utilized model organisms, a large portion of its genome remained uncharacterized. Computational prediction models will facilitate identification of novel gene functions. For instance, a recently initiated COMBREX project, the goal of which is to improve our understanding of microbial protein function by bridging computational and experimental approaches, chose *E. coli* as one of its two focus organisms (32). Network-based functional prediction tools, such as EcoliNet, will play key roles in such community-wide efforts. Expansion of our knowledge of pathways will contribute to better *E. coli* metabolic engineering. In addition, EcoliNet’s freely available functional gene associations can be used to reconstruct cofunctional gene networks for other bacterial species via orthology-based methods (31). Therefore, EcoliNet will be a useful research resource for not only *E. coli* but also other bacterial species.

## Funding

This work was supported by the National Research Foundation of Korea (2010-0017649, 2012M3A9B4028641, 2012M3A9C7050151) to I.L. Funding for open access charge: National Research Foundation of Korea (2010-0017649). 

*Conflict of Interest*: None declared.
